# Depression Preceding Diagnosis of Bipolar Disorder

**DOI:** 10.3389/fpsyt.2020.00500

**Published:** 2020-06-11

**Authors:** Claire O'Donovan, Martin Alda

**Affiliations:** Department of Psychiatry, Dalhousie University, Halifax, NS, Canada

**Keywords:** bipolar disorder, family history (FH), early onset, mixed depression, staging, adverse response to antidepressants, mood stabilizers, polarity at onset

## Abstract

This paper focuses on depression that precedes an onset of manifest bipolar disorder as early stage bipolar disorder. First, we review how to pragmatically identify the clinical characteristics of patients presenting with an episode of depression who subsequently go on to develop episodes of mania or hypomania. The existing literature shows a strong consensus: accurate identification of depression with early onset and recurrent course with multiple episodes, subthreshold hypomanic and/or mixed symptoms, and family history of bipolar disorder or completed suicide have been shown by multiple authors as signs pointing to bipolar diagnosis. This contrasts with relatively limited information available to guide management of such “pre-bipolar” (pre-declared bipolar) patients, especially those in the adult age range. Default assumption of unipolar depression at this stage carries significant risk. Antidepressants are still the most common pharmacological treatment used, but clinicians need to be aware of their potential harm. In some patients with unrecognized bipolar depression, antidepressants can not only produce switch to (hypo)mania, but also mixed symptoms, or worsening of depression with an increased risk of suicide. We review pragmatic management strategies in the literature beyond clinical guidelines that can be considered for this at-risk group encompassing the more recent child and adolescent literature. In the future, genetic research could make the early identification of bipolar depression easier by generating informative markers and polygenic risk scores.

## Introduction

Of people presenting with an episode of major depression, a certain proportion may in reality be suffering from depression that is of bipolar type. This could be for several reasons: 1) in many if not most cases bipolar disorder starts with symptoms of depression and first hypomania/mania may not appear until years later; 2) depression is considered a part of the bipolar genetic spectrum and thus some forms of depression are conceivably variants of bipolar disorder, particularly in those with a strong family history; and 3) previous episodes of hypomania or even mania may be missed in some patients.

In this paper we will deal with the first two possibilities acknowledging that we currently mostly misidentify first episode and early stage bipolar disorder (BD) when it presents as depression (“pre-bipolar depression” meaning depression before it is realized that it is bipolar by virtue of an ensuing hypo/manic episode) by defaulting to unipolar disorder. We will review the literature relevant for the best identification of depressive episodes in probable early stages of bipolar disorder that is prior to manifest (hypo) mania and suggest a pragmatic cost/benefit approach to management that handles uncertainty of final diagnosis to come.

## Polarity at Onset and Interval From First Depression to First Mania

Depressive episodes are common at the onset of bipolar disorder as shown in both retrospective and prospective studies. The proportion of those with a depressive index episode varies between different studies, but consistently is over 50% (range of 50%–80%). Family data suggest that the polarity at onset is possibly a heritable trait and may identify separate genetic subtypes of bipolar disorder (BD) (1).

The advantage of retrospective studies is usually their large sample size. On the other hand, they are prone to recall bias and especially less severe episodes are more difficult to remember. For this reason, prospective follow-up of young people at risk of bipolar disorder is valuable in mapping the early course more accurately. Repeated careful clinical assessments allow for detection of less severe mood states that might be overlooked in retrospect. Several such long-term cohort studies are described in the literature and practically all document high rates of depressive onsets. For instance, in the Canadian high-risk study major depression was reported as the initial episode in 58% of at-risk individuals and depressive disorder not otherwise specified (NOS) in another 19% while the remaining 23% of onsets were divided between mania, hypomania, and cyclothymia (2). Similarly, in the Dutch bipolar offspring cohort, 108 participants were followed for 12 years. At the second assessment, 15 out of 17 who had a diagnosis of bipolar disorder had an index episode of depression or dysthymia that preceded the first (hypo)mania by an average of 5.6 ± 4.4 years (3). In the Pittsburgh high-risk study, 25 out of 36 offspring diagnosed with bipolar disorder had a previous history of depression (4). And finally, several other high-risk studies that reported cross-sectional prevalence of mood disorders found especially high rates of depression in children of parents with bipolar disorder, reviewed by Vandeleur (5). See also a review of high-risk studies, prospective and retrospective by Vieta et al. (6).

Some studies suggest that there is also a difference in the manifestation of early depression depending on the family history. In the Pittsburgh high-risk study, those at risk of bipolar disorder had depression that was more severe with more prominent atypical symptoms, especially hypersomnia, fatigue, psychomotor retardation, and sense of hopelessness (7).

A related variable describing the early course of BD is the interval from first depression to mania or hypomania. This also tends to vary considerably both within and between studies. Thus, Berk et al. (8) reported an average of 7.6 ± 8.7 years and Cha et al. 5.6 ± 6.1 years (9). In both cases the interval is not only lengthy, but also highly variable between individuals. Murru et al. found the duration of untreated illness over 6 years on average and those with a longer untreated interval had more typically younger onset, predominantly depressive polarity, more chronic course, and more frequent comorbid conditions (10).

## Conversion From Major Depression to Bipolar Disorder

Another group of observations relate to longitudinal cohorts of people initially diagnosed with major depression. Given a sufficient length of follow-up, a certain proportion of them will convert to bipolar I or bipolar II disorder. The longest such observation comes from the Zurich cohort of Jules Angst. He and colleagues showed that after an initial somewhat steeper rate of conversion, from the 5^th^ year onward the proportion of those who developed a manic episode was about 1% per year and hypomanias occurred at a rate of 0.5% per year (11). Coryell et al. (12) observed conversion to mania and hypomania each in about 5% of patients previously diagnosed with depression and followed for 10 years. These rates are only marginally lower than the Angst data. In another long-term prospective cohort of patients with major depression followed for an average of 17.5 years, the rate of new onsets of mania was 7.5% and hypomania in 12.2%. The risk was associated with symptoms of psychosis and subthreshold hypomanic symptoms during the depressive episodes as well as early onset of depression (13). In a population cohort of 3012 young community participants, 3.6% of people with an initial depression were re-diagnosed with bipolar disorder after a period of up to 10 years, but the risk was substantially higher at 9% in those with onset of depression before the age of 17 (14). Finally, in a large population database study, the cumulative incidence of conversion was 8.4% over an average of 7.7 ± 5.4 years with the strongest predictors being the parental diagnosis of BD, psychotic depression, prior diagnosis of psychosis, and inpatient treatment for the index episode (15).

## Bipolar Depression in Absence of (Hypo)mania

However, not all with bipolar depression ever develop a manic episode. In some of them family history of BD is an indication that their depression is of a different type. Blacker and Tsuang estimated that about two-thirds of unipolar relatives of bipolar probands in fact had bipolar depression (16). It is to be noted that the distinction between this group and those that convert is somewhat artificial. The length of follow-up is clearly an issue—cf. the Zurich study in which some patients changed their diagnosis even after 50 years (11).

## Identification of Clinical Features That Can Help Identify Pre-Bipolar Depression

In the above sections we discussed the features of depressive episodes that, in retrospect, preceded a confirmed diagnosis of bipolar disorder or differentiated those with established diagnoses of BD versus unipolar depression. Clinicians, however, face a different task. They need to decide whether a patient presenting with depression might be suffering from bipolar depression and if so, what implications this has for clinical management.

There are no definitive criteria or biomarkers for depression preceding first episode hypo/mania and some would argue that it is unipolar depression until it converts. In this view, antidepressant induced switch is a helpful hint to alter course of treatment. The alternative view is that our goal is to better identify probable bipolar disorder at first/early episode depression, particularly in high risk families, cause no harm, and positively impact the long-term course of illness. In this latter approach, a staging strategy to enrich the data collected at a research level and a clinically useful cost/benefit strategy at a clinical level is imperative. One approach to depression at least in youth is to assign no polarity (unipolar or bipolar) until several episodes have occurred but this will not do justice to those who convert late or not at all.

**Table 1** provides a summary of clinical variables helpful in distinguishing unipolar and bipolar depression, using a triad of family history, course of illness, and symptoms.

**Table 1** also denotes those clinical features that are identifiable for the clinician relying on criteria as per Diagnostic and Statistical Manual (DSM).

**Table 1:** Potential predictors of bipolar course in comparison to unipolar course [see also (17–22)].

Symptoms	Course	Family History
Presence of mixed symptoms*	Early age of onset	Bipolar Disorder
Atypical depression*	Abrupt onset/offset	Suicide
Lability of mood	Frequent episodes	Severe mental illness
Psychomotor retardation*	Shorter episodes	
Pathological guilt	Post-partum episode	
Psychotic symptoms	Recurrent episodic	
Hyperthymic/cyclothymic temperament Catatonia*	Treatment resistance Seasonality*	
	Abrupt onset response to antidepressant/increase dose	

*These factors are present in some modified form in DSM V; the others require a broader understanding of the psychiatric literature.

## Assessment of Family History

Of this triad, the most robust predictor is family history of bipolar disorder, especially in early onset youth. Family history of completed suicide along with male sex are the only known predictors of completed suicide in this group (6). The most valuable course predictors are recurrence and early age of onset of depressive symptoms. The most consistent symptom-related factors are subsyndromal hypo/manic symptoms (mixed symptoms) and mood lability (6).

Family history, like recurrence and age of onset of first symptoms are readily available to the clinician who spends some time. It is often the first piece of information presenting to the treating clinician before any treatment commences. However, poor quality of family history information in routine clinical practice is a frequent critique in the literature (23, 24). While earlier studies endorsed under reporting of indirectly collected family history bipolar, the recent colloquialization of the term “bipolar” has led to concerns in the opposite direction (https://ibpf.org/articles/please-stop-saying-bipolar-when-you-mean-unpredictable-or-broken/accessed2020-02-16) (http://www.vh1.com/news/261723/bipolar-disorder-hollywood-misconceptions/accessed2020-02-16) (25).

Family study of individual family members with structured interviews is the most accurate method for research, with family history from a tool such as the Family History Research Diagnostic Criteria method (FH-RDC) from as many informants as possible providing a more accessible although less sensitive tool (26). Finally, there is some evidence for the Family History Screen which provides information on 15 psychiatric disorders including mania and takes 5–20 min, depending on complexity (27). **Table 2** provides clinical advice on verification of a patient report of family history bipolar disorder.

**Table 2:** Factors to consider in assessing family history of hypo/mania from an informant.

Is the person described a biological relative?
Has the person described adequate DSM criteria for hypo/mania?
Has the person described got persistent psychosis?
Is the behavioral manifestation apparent to family consistent with a probable diagnosis of hypo/mania
Has the person described been hospitalized?
Has the person received/responded to ECT?
Has the person described been prescribed lithium or divalproex or carbamazepine or lamotrigine?
Has the person described been prescribed higher dose atypical antipsychotics consistent with treatment of bipolar disorder?
Has the person described been prescribed antidepressant monotherapy long term and done well?
Has the person had serious impairment of function at some stage of their illness?
Has the person described ended their life by suicide or made a serious suicide attempt?

## Assessment of Course of Illness

There is little difficulty for the clinician in assessing early onset and recurrent episodic depression along with seasonality and hormonal status, and changes in the course of illness over time as long as attention is paid to past and prospective course in a methodical manner. This is imperative where there is early onset or a family history of bipolar disorder.

Pictorial graphing representation of the episodes can be particularly helpful and the patient can be engaged in the maintenance phase in depicting this (for example, see: https://bipolarnews.org/wp-content/uploads/2010/07/Patient-Retrospective-Manual.pdf accessed 04/27/2020). Unfortunately the use of pictorial graphing of episodes tends to be siloed in the bipolar disorder literature although it is helpful for both unipolar and bipolar illnesses and hence the pictorial representation of course tends to start with a hypo/manic episode and work backward. Staging models of bipolar disorder which identify depression as prodromal or non-specific or at increased risk of bipolar disorder (i.e. identify it as unipolar depression before it “converts”) are evolving and not currently yet of clinical help to the clinician's dilemma (28).

## Assessment of Mixed Symptoms

The assessment of mixed/hypomanic symptoms interspersed in a depressive episode is at best a challenge for the clinician when looking for guidance from the literature with both broad and narrow interpretations of mixed symptoms, variably defined in DSM and the broader literature, and a more specific unique mixed syndrome most notably propounded by Koukopoulos (29). DSM V mixed symptom specifier is the most restrictive diagnosis requiring three mixed hypomanic symptoms, excluding the overlapping symptoms of psychic and motor agitation, irritability, and distractibility (30). These above excluded symptoms, along with mood lability, the broader–irritability-anger-hostility continuum and an impulsive suicidality are considered the core of mixed state by many others (29, 31–33).

A broad assessment of DSM V criteria for major depression and hypo/mania along with mood lability and an irritability-anger –hostility factor without *a priori* assignment to pole may be most valuable to the clinician in assessing baseline and progression of illness state with or without treatment intervention. Of those tools available for assessment of mixed symptoms, the Zimmerman scale matches DSM V criteria for mixed symptom specifier (34).

Other continuous (between poles) assessment can be used such as using the Young Mania Rating Scale, along with the Montgomery-Asberg Depression Rating Scale (includes the irritability-hostility dimension but does not include lability or impulsivity (35, 36) and various structured interviews such as the Structured Clinical Interview for DSM or The Schedule for Affective Disorders and Schizophrenia).

There are a large number of other criteria and scales for mixed symptoms which variably include (37–39) and exclude the three overlapping DSM symptoms (39). They also include other symptoms and validate exclusion of some of the more frank manic symptoms of DSM mixed symptom specifier such as euphoria, less commonly found in mixed depression—see the Koukopoulos Mixed Depression Rating Scale (40).

Patients with manic symptoms can be more averse to reporting to the clinician and benefit from self-reporting scales of mania include the Altman scale and the Internal State scale (41, 42).The hypomania checklist (HCL 32) has been used specifically in major depression with mixed features (43). Sachs has published a combined DSM V clinical monitoring form (includes overlapping symptoms) and speaks to the need for careful and detailed assessment and follow-up (44, 45). Other scales include family history of bipolar disorder as a validating criterion, e.g. (46). Summary recommendations for assessment and monitoring are given in **Table 3**.

**Table 3:** Assessment and monitoring of major depression with family history of bipolar disorder or suicide.

Careful assessment and follow up of family history
Baseline and follow up monitoring of both manic and depressive symptoms
Weekly monitoring for 4 weeks
Bi-weekly monitoring up to 12 weeks
Use of collateral history
Specific attention to suicidality including impulsive suicidality
Specific attention to psychic or motor agitation
Specific attention to the irritability-hostility-aggression continuum
Follow course long term for alteration in course of illness
Referral to psychiatric services, specialty mood disorders clinics where available

## Pragmatic Management of Possible/Probable Pre-Bipolar Depression

There is now largely an acceptance that bipolar and unipolar depression are treated differently with overlap for a minority of bipolar I and some bipolar II depressions that may be pharmacogenetically distinct in response to antidepressants (47). It is accepted that bipolar depression with mixed features should not be treated with antidepressants (37, 47). What then of possible pre-bipolar depression/a depression with family history of bipolar disorder or early onset of depression with mixed symptoms or both? Recent clinical Practice Guidelines on major depression have been largely silent on the topic of potential bipolar depression, ignoring familial risk of bipolar disorder as a key consideration (48–51). The exception is the original Royal Australian Guidelines on mood disorders, a merger of unipolar and bipolar illnesses. By virtue of approaching the disorders in this unitary fashion, the management approach views the unipolar depressive episode as either unipolar or unknown bipolar, pays attention to differentiating symptoms and course factors and emphasizes family history of bipolar disorder as raising the “index of suspicion”. It emphasizes approaching the diagnosis as provisional to be clarified over time and acknowledges the possibility of iatrogenic worsening. It also emphasizes the close relationship between highly recurrent episodic depression and bipolar disorder which opens up the exploration for the treating psychiatrist that the episode of depression is unipolar but may respond pharmacologically similar to bipolar depression (52).

In contrast, recent treatment guidelines for children/adolescent depression are much more attentive to factoring family history of bipolar disorder into the assessment, gathering collateral history and providing detailed psychoeducation to families regarding potential bipolar outcomes (53, 54). There are several obvious reasons for this. Firstly, early age of onset is among the more solid predictors of bipolar depression and risk of manic switch in youth is 4.5 times greater than in adults (55). Mixed manic symptoms interspersed with depression are more common in youth (56). It is more firmly accepted that antidepressants can cause harm in adolescent depression with new onset of or increase in irritability, impulsivity and agitation, suicidal thinking, and behavior as well as the broader phenomenology of “activation” (53, 54, 57, 58). There are several long-term prospective cohorts of children with familial risk ongoing, cited above, with attempts to calculate risk of future bipolar course at least over the short term (59). Finally, the assessment of youth typically includes families which alters the perspective.

The more recent guidelines also reflect a significant number of recent studies and reviews in children and adolescents (and occasionally up to age 20) with major depression and family history of bipolar disorder summarized by Angal in a recent case report and review of the literature (56).

The Guidelines for Adolescent Depression in Primary Care (GLAD-PC) Toolkit published in 2018 keeps the clinician aware of potential future bipolar course, advise on frequency of careful monitoring in the first 12 weeks, emphasize supportive but active monitoring in shorter episodes, use of CBT/IPT in milder cases, low dose and slow titration (no increase for 4 weeks from fluoxetine 10 mg), avoidance of tricyclics which are ineffective, and avoidance of venlafaxine which has been associated with more self-harm events. (Guidelines for Adolescent Depression in Primary Care (GLAD-PC) Toolkit: http://www.gladpc.org accessed 2020-02-16).At the same time they make clear that severe episodes needed to be treated aggressively—the most challenging question for those with risk factors for bipolar depression is what pharmacological agent to use.

Some of these tools could be used in a similar fashion for monitoring those with family history of bipolar disorder regardless of age. However, even the youth guidelines do not adequately address the assessment of mixed symptoms in a depressive episode.

There is a need to accurately and thoroughly assess and monitor mixed symptoms particularly in those with major depression and family history positive for bipolar disorder both as initial presentation (pre-antidepressant) and in follow up monitoring. Quite apart from the known risk of manic/hypomanic switch, there is a small but significant literature on the association of antidepressants with worsening of depression, cycling of depression, emergent mixed symptoms, treatment resistance, and suicidality (not limited to youth) in a subgroup of depressed patients with and without potential mixed symptoms (22, 55, 60–66).

We retrospectively studied a small group of confirmed bipolar versus unipolar depressed patients (over 7 years) for earlier history. Family history of bipolar disorder and completed suicide predicted future bipolar course. Early onset depression as well as treatment emergent mixed symptoms, lability, psychomotor activation, and suicidality were significantly more common in the “pre-bipolar group” (60).

Antidepressant worsening is now increasingly accepted as a potential outcome in bipolar depression although still with considerable controversy about their efficacy and potential adverse effects and “tachyphylaxis” (63, 66–73). Serotonin reuptake inhibitors and bupropion are favored over more multimodal antidepressants in the International Society for Bipolar Disorders Guidelines on use of antidepressants in bipolar depression. Antidepressants are contraindicated in mixed bipolar depression and alternative mood stabilizing agents with antidepressant efficacy are considered rather than antidepressants (73).

Antidepressant worsening in child and adolescent depression is also accepted as a possibility in a minority of patients and antidepressants are held second line or for more severe cases and more frequently monitored than in adult guidelines The option of adding a mood stabilizer to the antidepressant is made explicitly in these recent guidelines (Guidelines for Adolescent Depression in Primary Care (GLAD-PC) Toolkit: http://www.gladpc.org accessed 2020-02-16).

Absent from any guideline is recommendation for a systematic assessment of baseline symptomatology and monitoring of outcome, whether “watchful waiting” monitoring the natural transition of illness, or with active specific pharmacological or psychotherapeutic intervention, particularly in relation to mixed symptoms. This can be as simple as assessing all DSM V symptoms of both depression and mania in a good clinical interview, but self-report tools noted above can be of benefit both for education and monitoring and allow also for collateral history for observable changes. The challenge is that there is no accepted definition of mixed symptoms.

## Specific Treatments for Depression With Family History of Bipolar Disorder

There can be no specific recommendations for this group at this time as all data is extrapolation. Clinicians should be experienced and have a good knowledge of unipolar, bipolar, child and adolescent, youth guidelines, and guidelines for mixed bipolar states and apply where they see fit and change according to treatment response (37, 47–54, 56, 57, 72, 73). Guidelines for first episode psychosis contain limited data on the depression with psychotic features group (6). Guidelines have unfortunately siloed data. For example, suggestions for “low dose—go slow” antidepressants in this group is largely borrowed from the child and adolescent guidelines (high risk group) and early psychosis literature (6).

Several recent papers, not reviewed here summarize the limited data on treatment of “unipolar “ depression with mixed features (33, 74–77) which may or may not proceed to bipolar disorder or in part have its own signature. As can be seen by the recency of papers quoted, up to date knowledge of the evolving literature is important as guidelines are already outdated.

**Table 4** outlines strategies to consider in approaching major depression, family history bipolar disorder—of most importance in youth but remaining important in young adults all the way to old age. These are extrapolated from both unipolar and bipolar guidelines above where the agent is recommended as effective in both unipolar and bipolar depression.

**Table 4:** Treatment considerations for major depression with family history of bipolar disorder or suicide.

Consideration of supportive therapy, CBT, IPT, and FFT in milder, shorter depression
Antidepressants may cause harm in this group
Cautious low dose—go slow strategy for antidepressants if used.
Consider lithium especially in those with family history response to lithium/family history suicide
Consider avoiding SNRIs and TCAs
Consider tapering antidepressant if mixed symptoms evolve
Consider adding mood stabilizer
Consider neuromodulation (ECT and with consequent personalized treatment rTMS) in moderate to severe especially mixed depression
Consider lamotrigine or quetiapine as they are used in both unipolar and bipolar depression
Consider ziprasidone or lurasidone augmentation if mixed symptoms

CBT, cognitive behavioral therapy; IPT, Interpersonal Therapy; FFT, Family Focused Therapy; SNRIs, serotonin-norepinephrine reuptake inhibitors; TCAs, tricyclic antidepressants; ECT, electroconvulsive therapy; rTMS, repetitive transcranial magnetic stimulation.

Antidepressants continue to have a presence as indeed some of these cases may have little diathesis to bipolar outcome or respond to antidepressants effectively as do a minority of those with known bipolar depression. The potential *post hoc* fallacy assuming that an antidepressant has been effective has to be balanced against the presumed natural duration of a depressive episode which in pre-bipolar depression can be quite short with abrupt offset. Careful delineation of baseline symptoms of both poles will help identify natural evolution of mixed symptoms or indeed antidepressant worsening, even if preceded by a honeymoon response. The principle of *primum non nocere* is most challenging with antidepressants but the use of potent psychotropics of any kind has to be balanced against the possible benefits of evidence based psychotherapies particularly in milder or earlier onset cases. Inevitably many cases will be severe, and we point to agents, pharmacological and neuromodulatory, that have benefit and/or less harm in either unipolar or bipolar depression either in mono or combined therapy such as quetiapine, lamotrigine and lithium, and electroconvulsive therapy. Repetitive transcranial magnetic stimulation has evidence in both unipolar and bipolar depression but there is report of a very small number of cases of treatment emergent hypo/mania (78). We would urge caution in extrapolating treatment recommendations from DSM IV diagnosed mixed mood which is directed to a mixed manic syndrome rather than depression with some mixed symptoms. Ziprasidone and lurasidone have limited data in mixed depression (35, 36, 77).

Lithium should be considered as a serious contender given its benefit in both recurrent episodic depression and bipolar depression, its neuroprotective effects, and its antisuicidal effects, most especially but not exclusively in those with a family history of lithium response or completed suicide. It has no specific evidence in depression with mixed symptoms, but there appears to be a good case for its use (79). The choice here should be considered in light of proposed predictors of lithium response (family history, especially of lithium-responsive illness, absence of mood incongruent psychosis, and low rates of comorbid conditions (80).

## Conclusions and Future Directions

The existing literature supports the distinction between depression that is likely a manifestation of bipolar disorder and depressive disorder. The most consistent features helpful in such differentiation include early onset, highly recurrent clinical course, family history of bipolar disorder and/or completed suicide, and adverse response to antidepressants. Arguably, these factors are not infallible. It is hoped that in the future, laboratory or brain imaging technologies can contribute further to more accurate diagnosis. One such measure could be based on genome-wide genetic data. It is conceivable that, as more genetic markers of bipolar disorder and depression are discovered, it will be possible to differentiate the subtypes of depression by, for instance, polygenic risk scores. These could be constructed specifically with the goal of capturing genetic differences between unipolar and bipolar depression rather than polygenic scores for each condition alone. Recently, Liebers et al. analyzed polygenic risk score (PRS) differences between UD and BD in 843 BD and 930 UD patients (81). Those with BD had higher PRS for BD and schizophrenia, but both contributed modestly to the overall classification (AUC of 0.64 for either PRS) and only minimally once the authors used clinical profiles for classification. Nevertheless, large datasets are becoming now available to analyze genetic differences between the two types of depression further, see for instance the recent large scale analysis by Coleman et al. (82). How practical such an approach will be remains to be seen. On the one hand, genome-wide analyses point to a significant correlation in genetic liability to major depression and bipolar disorder, on the other hand, the clinical differences give hope that their differentiation at the genetic level should be also possible.

## Author Contributions

CO'D and MA both reviewed the relevant literature and drafted and revised the manuscript.

## Conflict of Interest

The authors declare that the research was conducted in the absence of any commercial or financial relationships that could be construed as a potential conflict of interest.
